# Effect of post-mortem delay on N-terminal huntingtin protein fragments in human control and Huntington disease brain lysates

**DOI:** 10.1371/journal.pone.0178556

**Published:** 2017-06-01

**Authors:** Menno H. Schut, Stefano Patassini, Eric H. Kim, Jocelyn Bullock, Henry J. Waldvogel, Richard L. M. Faull, Barry A. Pepers, Johan T. den Dunnen, Gert-Jan B. van Ommen, Willeke M. C. van Roon-Mom

**Affiliations:** 1 Department of Human Genetics, Leiden University Medical Center, Leiden, The Netherlands; 2 Centre for Brain Research and Department of Anatomy with Radiology, University of Auckland, Auckland, New Zealand; 3 Leiden Genome Technology Center, Leiden University Medical Center, Leiden, The Netherlands; Hokkaido Daigaku, JAPAN

## Abstract

Huntington disease is associated with elongation of a CAG repeat in the *HTT* gene that results in a mutant huntingtin protein. Several studies have implicated N-terminal huntingtin protein fragments in Huntington disease pathogenesis. Ideally, these fragments are studied in human brain tissue. However, the use of human brain tissue comes with certain unavoidable variables such as post mortem delay, artefacts from freeze-thaw cycles and subject-to-subject variation. Knowledge on how these variables might affect N-terminal huntingtin protein fragments in post mortem human brain is important for a proper interpretation of study results. The effect of post mortem delay on protein in human brain is known to vary depending on the protein of interest. In the present study, we have assessed the effect of post mortem delay on N-terminal huntingtin protein fragments using western blot. We mimicked post mortem delay in one individual control case and one individual Huntington disease case with low initial post mortem delay. The influence of subject-to-subject variation on N-terminal huntingtin fragments was assessed in human cortex and human striatum using two cohorts of control and Huntington disease subjects. Our results show that effects of post mortem delay on N-terminal huntingtin protein fragments are minor in our individual subjects. Additionally, one freeze-thaw cycle decreases the huntingtin western blot signal intensity in the cortex control subject, but does not introduce additional N-terminal huntingtin fragments. Our results suggest that subject-to-subject variation contributes more to variability in N-terminal huntingtin fragments than post mortem delay.

## Introduction

Huntington disease (HD) is an autosomal dominant neurodegenerative disease that primarily affects the striatum and is caused by a CAG repeat expansion in the first exon of the *HTT* gene [1]. This results in a toxic huntingtin protein (htt) with an N-terminally expanded polyQ-stretch [2, 3]. Subsequent studies with HD mouse models with comparable polyQ-repeats expressing either N-terminal, or full length htt showed that N-terminal htt fragments invoke a more severe phenotype [4–6]. Smaller fragments induce a more severe phenotype [7–9]. In addition, toxicity of mutant N-terminal fragments could be increased by modifications such as loss of the first 17 amino acid stretch preceding the polyQ repeat [10]. The htt N-terminal domain contains several proteolytic cleavage sites, some of which have been linked to HD pathology [11–14]. Results obtained from different mouse-models are sometimes conflicting. YAC128 mouse models expressing full length mutant htt resistant against caspase-3 or caspase-6 cleavage suggested that caspase-6 cleavage at amino acid 586 contributes more to HD pathogenesis than caspase-3 cleavage at amino acids 513 and 552 [15]. Murine expression of the N552-htt fragment was shown to be more lethal compared with other caspase-associated htt fragments including N586-htt [8]. With regards to HD research on N-terminal htt protein fragments, it is important to note that studies in rat, mouse and post mortem human brain tissue have indicated that calpain-mediated proteolysis also occurs during post mortem delay (PMD) which is the time between death and tissue preservation. In rat brain, PMD-related fodrin calpain cleavage fragments were observed [16], and in mouse and human brain tissue PMD-related GSK-3 truncation by calpain was demonstrated [17]. Hence, it is important to distinguish biologically relevant N-terminal htt fragments from those that are formed during PMD. Available data on N-terminal htt protein fragments in human post mortem brain is limited, but also indicates a role in HD pathology. N-terminal htt fragments in post mortem human HD striatum differ with post mortem human control striatum [18]. Also, the N552-htt fragment, associated with HD pathology in a HD mouse model, was detected in post mortem human HD and control brain tissue lysates [13]. Therapeutic strategies involving prevention of formation of N-terminal htt fragments are currently pursued [19]. However, extrapolation of results obtained in cell and animal models to the human brain is difficult. N-terminal htt fragments may vary between different biological systems [20]. Furthermore, research involving post mortem human brain tissue involves unavoidable variables that are difficult to control such as PMD, post mortem processing, and interpersonal variation. Effects of PMD on biological parameters have been well described [21]. In rat brain, synaptic density and vesicles decline after a PMD of 15hr, with subtle changes on synaptic structure [22]. Binding sites for forskolin, an activator for adenylate cyclase, were already reduced in rat striatum after a PMD of 4 hours [23]. PMD related effects vary between different molecules and proteins. No effect of PMD for up to 72 hours was observed for fatty acid molecules in post mortem human brain tissue [24]. PMD was shown to variously affect different proteins in mouse CNS [25]. A study on different post mortem human brain samples indicated that levels of synaptic proteins PSD-95 and syntaxin, but not synaptophysin, decline with PMD [26]. However, care must be taken when comparing different subjects with comparable PMD because PMD related effects might also vary between subjects [27, 28]. In the current study, we have assessed effects of PMD, freezing and inter-individual variation between human subjects on the profile of N-terminal htt fragments on western blot. Our study shows a small effect of artificially induced PMD on N-terminal htt fragments in cortex tissue from one control subject, and in striatal tissue from one HD subject. Furthermore, one freeze-thaw cycle already adversely affected the western blot signal for huntingtin in the cortex tissue from the control subject, but did not result in the appearance of novel N-terminal htt fragments. Finally, analysis of nine control versus nine HD subjects matched for age, gender and PMD showed that there was more inter-individual variability in the profile of N-terminal htt fragments than the variability introduced by PMD.

## Materials and methods

### Human brain tissue

Post mortem human cortex and striatal brain tissue from control and HD subjects was obtained from the Neurological Foundation of New Zealand Human Brain Bank, Centre for Brain Research, University of Auckland. The temporal cortex tissue was obtained 1 hour after surgery on a patient suffering from severe epilepsy. Tissue was transported at 4°C. Tissue was obtained with the approval by the University of Auckland Human Participants Ethics Committee. Informed written consent was obtained in all cases. None of the donors were from a vulnerable population and all donors or next of kin provided written informed consent that was freely given. See also the website of the Neurological Foundation of New Zealand Human Brain Bank for more information: http://neurological.org.nz/what-we-do/human-brain-bank. See Table 1 for the complete list of samples with clinical information.

**Table 1:** Clinical information.

Type	Number	Name	Age	Gender	PMD	CAG 1	CAG 2	Grade
HD	n.a.	HC107	75	M	3	43	19	3
Control	C1	H110	83	F	14	N.A.	N.A.	n.a.
Control	C2	H202	83	M	14	N.A.	N.A.	n.a.
Control	C3	H157	66	M	15	N.A.	N.A.	n.a.
Control	C4	H155	61	M	7	N.A.	N.A.	n.a.
Control	C5	H146	61	M	15	N.A.	N.A.	n.a.
Control	C6	H159	53	M	16.5	N.A.	N.A.	n.a.
Control	C7	H174	59	M	24.5	N.A.	N.A.	n.a.
Control	C8	H130	32	M	13	N.A.	N.A.	n.a.
Control	C9	H200	56	M	23	N.A.	N.A.	n.a.
HD	HD1	HC111	91	F	18	40	15	2
HD	HD2	HC137	83	M	13	41	17	1
HD	HD3	HC133	65	M	14	43	17	3
HD	HD4	HC134	62	M	9	43	18	2
HD	HD5	HC113	58	M	14	44	28	2
HD	HD6	HC120	51	M	15	46	10	2
HD	HD7	HC115	56	M	16	46	16	2
HD	HD8	HC132	32	M	14	47	17	1
HD	HD9	HC119	51	M	15.5	48	17	3

Age: Age in years at death. F: female, M: male. PMD: post mortem delay in hours. CAG1 = CAG repeat length of mutant allele, CAG2 = CAG repeat length of normal allele. N.A. Not assessed. n.a. Not applicable. HD grade according to [29]. Average age for control subjects = 61.6 ± 15.5 years, HD subjects = 61.0 ± 17.6 years. Average PMD for control subjects = 15.8 ± 5.3 hours, HD subjects = 14.3 ± 2.5 hours. Average CAG repeat length in HD subjects = 44.2 ± 2.7 CAGs (mutant allele), 17.2 ± 4.7 CAGs (normal allele).

### Human brain tissue for analysis of post mortem delay effects

To mimic post mortem delay, tissue samples were taken from the main tissue specimen at regular time intervals (sampling). During sampling, tissue was left at room-temperature under sterile conditions. 1 ml of chilled homogenization buffer (150 mM Sucrose, 15 mM HEPES pH 7.9, 60 mM KCl, 0.5 mM EDTA pH 8.0, 0.1 mM EGTA pH 8.0, 1% Triton X-100) was added. Tissue was homogenized with a bullet blender (Next Advance) for 3 minutes, strength 8 using 0.5 mm stainless steel beads. Homogenized tissue was kept on ice for 1 hour and centrifuged cold at full speed for 10 minutes. Supernatant was aliquoted in 100 μl portions, snap-frozen and stored at -80°C. To mimic post mortem delay with and without freeze-thaw cycle, the temporal lobe tissue was divided into two halves immediately after obtaining the tissue. One half was subsequently sampled, while the other was snap-frozen and stored at -80°C according to [30] for 11 days before sampling.

### Post mortem human brain tissue lysates

For every subject, sensory/motor cortex and caudate nucleus tissue was collected separately. Per subject and region, 15 slides (thickness: 30 μm) of unfixed tissue were collected on glass slides with a cryostat-microtome (LEICA). Grey matter was scraped off, weighed and collected in 0.5 ml eppendorf tubes. 10 μl of chilled homogenization buffer was added per μg of tissue with a minimum of 200 μl. Tissue was homogenized with a bullet blender (Next Advance) for 3 minutes, strength 8 using 0.5 mm stainless steel beads. Homogenized tissue was kept on ice for 1 hour, centrifuged cold at full speed for 10 minutes. Supernatant was aliquoted in 100 μl portions, snap-frozen and stored at -80°C.

### Western blotting

100 μg of human brain lysate was used per sample. To detect full length htt, proteins were separated by SDS-PAGE according to the “shorter CAG repeats” protocol [31]. Proteins were transferred to a nitrocellulose membrane (Trans-Blot Turbo Transfer Pack Midi #170–4158, Bio-Rad, Hercules CA, USA) using the Trans-blot Turbo Transfer system (BioRad) with settings 2.5 A for 10 minutes. Blots were blocked with TBS containing 5% (w/v) non-fat milk (Nutricia, Schiphol, The Netherlands), and incubated with primary antibody 3702–1 (Epitomics, Burlinggame CA, USA) that binds the htt N17 terminus [32]. Secondary antibody goat anti rabbit IRDye800 (LI-COR, Lincoln, USA) diluted 1:5000 in TBS containing 5% non-fat milk (w/v). Blots were analyzed with the Odyssey infrared imaging system and Odyssey software version 3.0 (LI-COR). To detect htt fragments, proteins were separated by SDS-PAGE by running at 40 mA constant through the stacking gel and at 50 mA constant through the 10% separating gel alongside a protein size marker (PageRuler, Thermo Fisher, St Leon-Rot, Germany). Proteins were blotted onto either a nitrocellulose membrane for Control versus HD subjects (Trans-Blot Turbo Transfer Pack Midi #170–4158, Bio-Rad, Hercules CA, USA), or a PVDF-membrane for post mortem delay samples (Trans-Blot Turbo Transfer Pack Midi #170–4157), using the Trans-Blot Turbo Transfer System (Bio-Rad) with settings 1.0 A for 30 minutes. Blots were blocked with TBS containing 5% non-fat milk (Nutricia). For nitrocellulose-blots, primary incubation was performed with antibodies 3702–1 (Epitomics) and mouse anti β-actin. Secondary incubation was performed with goat anti rabbit IRDye800 (LI-COR) and goat anti mouse IRDye680 (LI-COR). All antibodies were diluted 1:5000 in TBS containing 5% non-fat milk (Nutricia). Blots were analysed with the Odyssey reader and viewed using the Odyssey software version 3.0 (LI-COR) with the linear manual sensitivity set at 5, or 6 if blot was viewed at “high sensitivity”. For PVDF-blots, primary incubation was performed with antibody 3702–1 (Epitomics) diluted 1:1000. Secondary incubation was performed with a Goat anti Rabbit antibody conjugated with Horse Radish Peroxidase (Santa Cruz) diluted 1:10.000. Blots were visualized using ECL+ substrate (#32132, ThermoFisher Scientific), and Hyperfilm ECL (#28906837, GE healthcare). For the β-actin loading control, blots were stripped, re-blocked and incubated with mouse β-actin diluted 1:1000, followed by a Goat anti Mouse antibody conjugated with Horse Radish Peroxidase (Santa Cruz) diluted 1:10.000. Densitometric analysis was performed with image J. Intensities of relevant bands were reported as percentages of the total htt signal within the associated lane. Values for the median and quartiles were calculated in Microsoft excel 2010 with the “MEDIAN” and “QUARTILE.EXC” commands.

## Results

### Two wild-type N-terminal htt fragments increase with artificial PMD in cortex temporal lobe tissue from an individual subject

**Fig 1:**
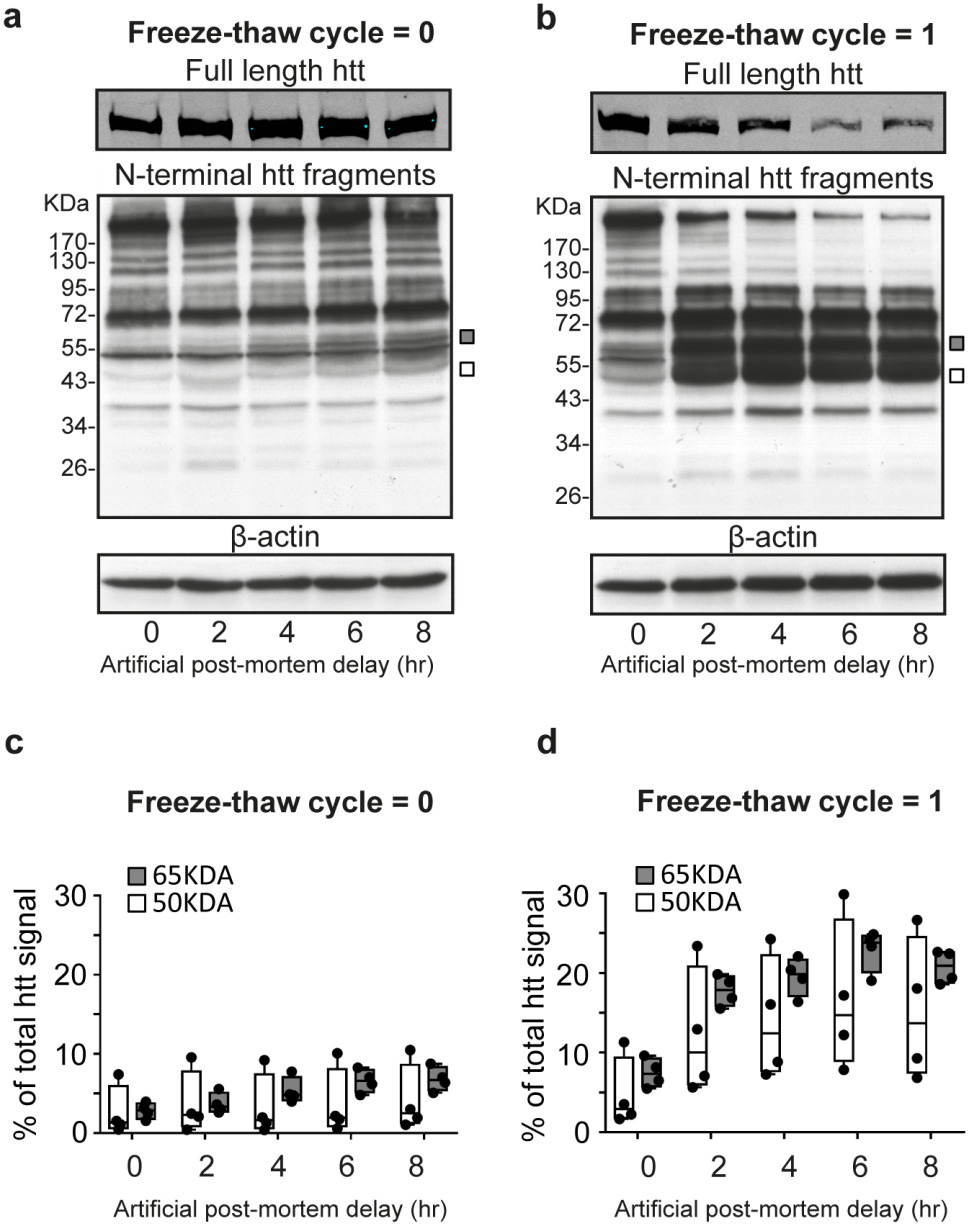
N-terminal huntingtin fragments in control temporal cortex tissue from an individual subject. Western blot analysis of temporal lobe tissue samples from tissue with no (A) and one freeze-thaw cycle (B) before sampling. Upper blot: full length htt. Middle and lower blot: N-terminal htt fragments with β-actin loading control. Squares: position of bands that increase with increasing artificial PMD (50kDa = white, 65kDa = grey). kDa = Molecular weight in kilodaltons, Artificial post mortem delay in hours (hr). (C) Whisker boxplot showing image J quantification of PMD-related htt bands at indicated timepoints relative to total htt (0 freeze-thaw cycli). (D) Whisker boxplot showing image J quantification of PMD-related htt bands at indicated timepoints relative to total htt (1 freeze-thaw cyclus). Boxes indicate the first and third quartile around the median (50 kDa = white, 65kDa = grey). Data points are shown as dots and represent four technical replicas of the same sample.

To assess the effect of PMD on N-terminal htt fragments, we obtained human cortex temporal lobe tissue (cortex) one hour post-surgery. One half was stored at -80°C and the other half was sampled at regular time-intervals to mimic PMD (artificial PMD). Using western blot analysis with an antibody that binds huntingtin at the N-terminal end, we observed two N-terminal htt fragments (of 50kDa and 65 kDa respectively) associated with artificial PMD. For the 50 kDa fragment, the data shows a larger spread with respect to the 65 kDa fragment. We detected no change in the full length htt signal between time points T = 0 hours (T^0hr^) and T = 8 hours (T^8hr^) for tissue that did not underwent one freeze-thaw cycle (Fig 1A). A comparison between T^0hr^ and T^8hr^ band intensities shows a slight increasing trend for the 50 kDa htt N-terminal fragment (median T^0hr^ = 1.32%, Q1 = 0.63% / Q3 = 5.94% versus median T^8hr^ = 2.47%, Q1 = 1.28% / Q3 = 8.62%). For the 65 kDa fragment, the difference in band intensity between T^0hr^ and T^8hr^ is more pronounced albeit still relatively small (median T^0hr^ = 2.85%, Q1 = 1.82% / Q3 = 3.76% versus median T^8hr^ = 6.71%, Q1 = 5.41% / Q3 = 8.34%). Next, we performed the same experiment using the frozen half of the cortex temporal lobe tissue sample. Here, we detected N-terminal fragments of the same molecular weight compared with the non-frozen tissue, but western blot bands corresponding to full-length and N-terminal htt fragments of more than 100 kDa became weaker with increasing artificial PMD (Fig 1B). The relative intensities of the 50kDa and 65kDa bands at T^0hr^ from the frozen sample were higher compared to the same bands detected in the non-frozen tissue (Fig 1A and 1B). In addition, the difference between T^0hr^ and T^8hr^ for both N-terminal fragments was larger when compared with the non-frozen tissue. N-terminal htt fragment of 50 kDa: median T^0hr^ = 2.88%, Q1 = 1.81% / Q3 = 9.37% versus median T^8hr^ = 13.68%, Q1 = 7.46% / Q3 = 24.52%. N-terminal htt fragment of 65 kDa: median T^0hr^ = 7.32%, Q1 = 5.74% / Q3 = 9.25% versus median T^8hr^ = 20.91%, Q1 = 18.79% / Q3 = 22.58%. Furthermore, this increase in band intensity was already apparent at T^2hr^ for both fragments (50 kDa: median T^2hr^ = 10.01%, Q1 = 5.99% / Q3 = 20.78% and 65 kDa: median T^2hr^ = 17.85%, Q1 = 15.86% / Q3 = 19.59%). This is in contrast to the subtle gradual increase observed for these N-terminal htt fragments in the non-frozen tissue. We did not detect additional N-terminal htt fragments that were introduced by the freeze-thaw cycle. Although, this result was obtained from one individual subject, it indicates that freezing of brain tissue affects htt signal strength, but not the profile of htt protein fragments in control cortex brain tissue samples.

### Artificial PMD introduces N-terminal htt fragments in HD striatal tissue from an individual subject

**Fig 2:**
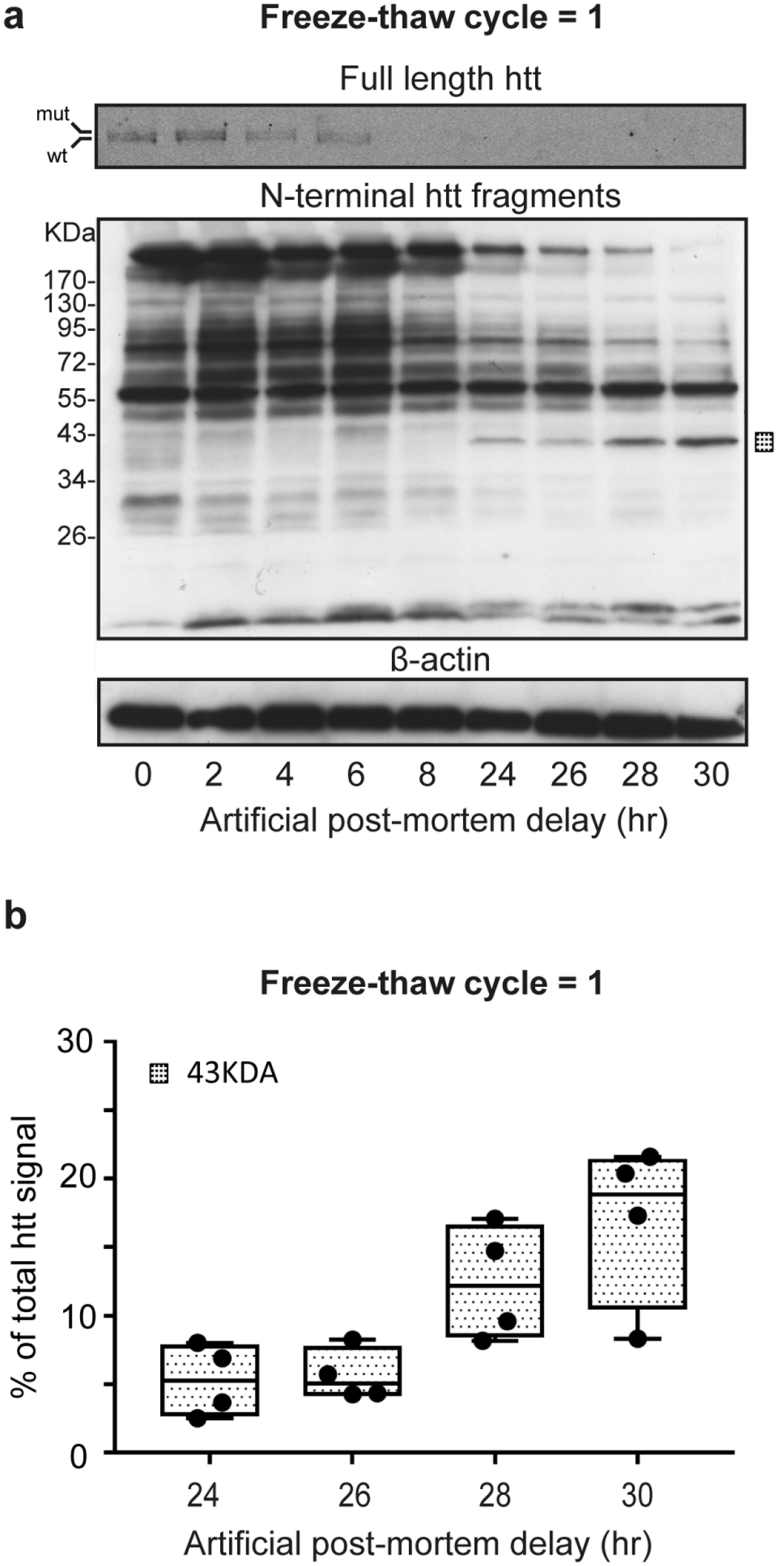
Effect of artificial post mortem delay on N-terminal huntingtin fragments in post mortem HD striatal tissue from an individual subject. (A) Western blot analysis of HD striatal tissue (1 freeze-thaw cyclus). Upper blot: full length htt. Middle and lower blot: N-terminal htt fragments with β-actin loading control. Square: Band increased with increasing artificial PMD. kDa = Molecular weight in kilodaltons, Artificial post mortem delay in hours (hr). (B) Whisker boxplot showing image J quantification of the 43kDa band at indicated timepoints relative to total htt. Boxes indicate the first and third quartile around the median. Data points are shown as dots and represent four technical replicas of the same sample. mut = full length mutant htt, wt = full length wild-type htt.

We obtained striatal tissue that was stored -80°C from an individual HD subject with a short initial PMD of (3 hours). Using this tissue, we analyzed the effect of an increasing artificial PMD in human HD striatal tissue. Careful examination of the western blot analysis of full length htt reveals two bands corresponding to wild-type and mutant huntingtin (e.g. Fig 2A timepoints 2 hr and 4 hr). This is in agreement with findings in human HD fibroblast lysates [31]. Artificial PMD decreased band intensities of full-length htt and most N-terminal htt fragments in HD striatal tissue. However, we observed a band at 43 kDa that appeared after an artificial PMD of 24hr (Fig 2A). Additionally, we were able to detect a band corresponding to a small N-terminal htt fragment (<26 kDa) that increased after 24hr, but this low molecular weight band could not reliably be quantified. ImageJ analysis of the 43 kDa band (Fig 2B) revealed an increase of band intensity at T^30hr^ compared with T^24hr^ (Median T^24hr^ = 5.30%, Q1 = 2.83% / Q3 = 7.75% versus median T^30hr^ = 18.85%, Q1 = 10.58% / Q3 = 21.30%. Hence, in our HD subject, the 43 kDa N-terminal htt fragment is associated with longer artificial PMD.

### Huntington disease subjects show greater variation in full-length and N-terminal huntingtin protein fragment profiles than control subjects

**Fig 3:**
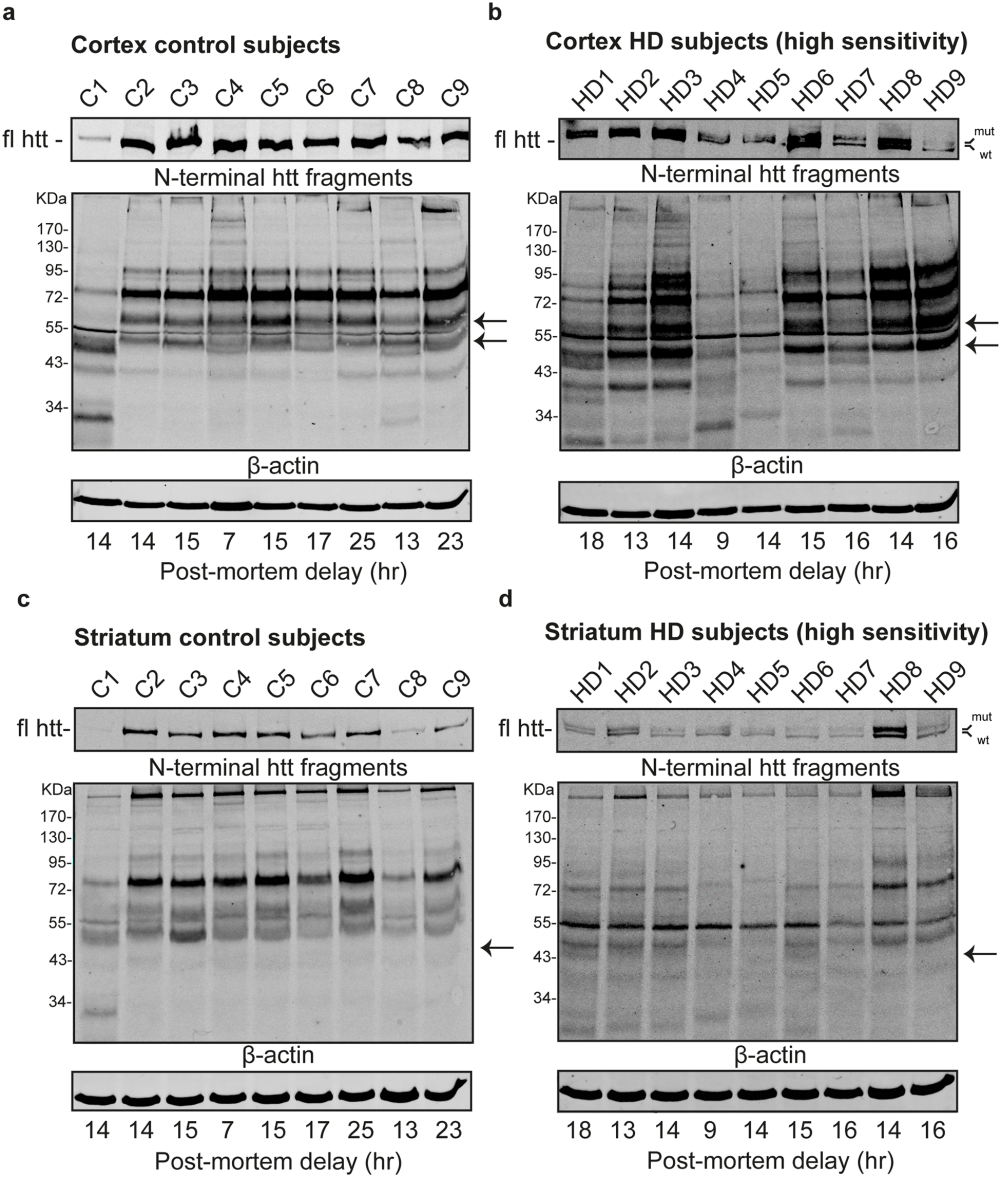
Comparison of N-terminal huntingtin fragments in human post mortem control and HD cortex and striatum tissue. Western blot analysis of (A) Cortex control subjects, (B) Cortex HD subjects, (C) Striatum control subjects and (D) Striatum HD subjects. Control and HD subjects were age, gender and post mortem delay matched. Upper blot: full length htt (fl htt). Middle and lower blot: N-terminal htt fragments with β-actin loading control. Arrows: position of bands associated with a post mortem delay related increase in intensity. kDa = Molecular weight in kilodaltons, Post mortem delay in hours (hr). High sensitivity: Blot analyzed at a higher viewing-sensitivity. mut = full length mutant htt, wt = full length wild-type htt.

Having identified N-terminal htt fragments in individual subjects that could be attributed to longer PMDs, we next analyzed N-terminal htt fragments in post mortem tissue in nine human control subjects (mean age = 61.6 ± 15.5 years, mean PMD = 15.8 ± 5.3 hours) and nine human HD subjects (mean age = 61.0 ± 17.6 years, mean PMD = 14.3 ± 2.5 hours). We observed that the western blot signal for full-length htt and N-terminal htt fragments was stronger in control subjects compared to HD subjects, and overall stronger in cortex tissue compared to striatal tissue, while no differences in the β-actin loading controls were observed (S1 Fig). Therefore, for optimal comparison, western blot results for the HD subjects are shown with the odyssey software configured at a higher viewing-sensitivity. In control cortex tissue, results were similar for subjects C2-C9 showing a consistent banding pattern with subtle interpersonal differences. The band-pattern for subject C1 was very different, showing relatively more N-terminal htt fragments below 55 kDa (Fig 3A). In HD cortex tissue (Fig 3B), interpersonal variation was more prominent and subjects HD1, HD4 and HD5 showed a weaker N-terminal htt profile. Smaller N-terminal htt fragments below 55 kDa appeared more pronounced with respect to the total htt signal in HD cortex tissue compared with control cortex tissue (S1 Fig). For both control and HD cortex subjects, western blot bands for N-terminal htt fragments were most prominent between 43 kDa and 95 kDa. This was in concordance with the N-terminal htt fragment profile induced by the artificial PMD of > 2 hours in temporal lobe tissue from the individual control subject stored at -80°C (Fig 1B). In addition, the N-terminal htt fragments of 65kDa and 50kDa that were associated with PMD in the individual control subject temporal lobe tissue were also detected in both control and HD cortex tissue (Fig 1B versus Fig 3A and 3B). This suggests these bands are non-specific cleavage products. No correlation between the PMD of the various subjects and band intensity for the 50kDa and 65kDa N-terminal htt fragments was observed. A possible explanation for this lack of correlation is that all subjects have a PMD of > than 8hr, and the relative intensity of the 50kDa and 65kDa bands does not markedly differ between time points of 2 hours or larger (Fig 1B and S2 Fig). In addition, we also detected the 43 kDa N-terminal htt band associated with PMD in the individual HD subject in the control and HD cortex tissue (Fig 2 versus Fig 3A and 3B).

Next, we performed western blot on post mortem striatal tissue from the same control and HD subjects (Fig 3C and 3D). Between control subjects (Fig 3C) the N-terminal htt fragment profile was similar, but we observed some interpersonal variation in signal intensity. The overall htt western blot signal in striatum was weak for all HD subjects (Fig 3D and S1 Fig). Between HD subjects the N-terminal htt fragment profile was similar with small interpersonal variation. Comparison between control and HD subjects for striatum revealed that the most prominent N-terminal htt fragment was detected at 80kDa in most control subjects, while in most HD subjects (HD1-HD6) the most prominent N-terminal htt fragment was detected at 55kDa. In subjects HD8-HD9, both 80kDa and 55kDa N-terminal htt fragments were detected equally. For control subjects, N-terminal htt fragments of similar sizes can be observed in both cortex and striatal tissue (Fig 3A and 3C). On the other hand, in HD subjects the 55kDa N-terminal fragment is less prominent in cortex tissue compared with striatum (Fig 3B and 3D).

## Discussion

In the current study we have examined PMD effects on N-terminal htt protein fragments in human brain tissue using western blot analysis on temporal lobe tissue obtained directly post-surgery from a control human subject. Our results show that in our control subject, increasing PMD resulted in a gradual increase of N-terminal htt fragment bands between 50 kDa and 65 kDa on western blot. Other studies detected a similar N-terminal htt fragment of 50 kDa in post mortem human cortex brain tissue obtained from control subjects with a PMD of 9 to 16 hour [18, 20]. Although we were only able to perform our study on one subject, our results indicate that this 50kDa N-terminal htt fragment could be associated with PMD. This, because a 50kDa and 65kDa band could also consistently be detected in cortical tissue from 18 subjects (9 control and 9 HD) with a PMD of 8 hours or greater. The addition of a freeze-thaw cycle increases the chance of reliably detecting these 50kDa or 65kDa bands. Other N-terminal htt fragments detected in [18, 20], and the caspase-3 related N-terminal htt fragment [13] detected in the cortex of human control subjects were not detected in our study. Although our findings do not fully exclude an association between PMD and these N-terminal htt fragments in as PMD effects might vary between different subjects, our results do suggest that there is no association between PMD and the N-terminal htt fragments reported in [13, 18, 20]. Our results on the cortex temporal lobe tissue sample also indicate that the effect of one freeze-thaw cycle on N-terminal htt fragments is quantitative, but not qualitative. We observed that one freeze-thaw cycle reduced the htt signal, especially for larger (>95 kDa) htt fragments and the full-length protein. Furthermore, PMD-related N-terminal htt fragments at 50kDa and 65kDa appeared at earlier time points for tissue that underwent one freeze-thaw cycle, suggesting their formation is enhanced due to freeze-thawing. However, no additional N-terminal htt fragments were observed due to freeze-thaw effects. Although we were not able to perform the freeze-thaw experiments for more than one subjects due to practical constraints, our results indicate that the common practice of tissue storage at -80°C and subsequent re-thawing for use is not expected to greatly influence study results on N-terminal htt fragments. This is an important finding because there is a well-established link between HD-pathology and small N-terminal htt fragments [7, 13]. Our experiments showed that artificial PMD gave rise to different N-terminal htt fragments in post mortem control temporal lobe tissue when compared with post mortem HD striatal tissue. This could suggest that PMD differently affects HD and control brains or different brain regions, but this difference might also be due to interpersonal differences between both subjects. The results on striatal tissue suggest that the 43 kDa band that is associated with PMD in HD striatum, appears between 8 and 24 hours. A striatal 43 kDa N-terminal fragment was reported before in HD striatum with PMD’s of 9 to 16 hours [18]. In our cortex control and HD subjects, we observed inter individual differences in N-terminal htt fragments, especially for HD subjects. However, we did not observe a correlation between N-terminal htt bands and the PMD’s of individual subjects. For the cortex control subjects this is most likely due because all subjects had a PMD of around 8 hours or larger. The relative abundances of the PMD-associated 50 kDa and 65 kDa htt fragments between T = 2, T = 4, T = 6 and T = 8 were comparable in our subject. Hence, variance in relative abundance of these fragments between subjects with a PMD of >8hr is not expected to be PMD-associated. We did not observe the PMD-associated 43 kDa N-terminal in our striatal HD subjects. Possibly, this was due to the weaker western blot signal for these subjects. It is likely that the observed inter individual differences between subjects are mainly caused by other factors such as genetic or environmental factors. We did not observe different N-terminal htt fragment profiles between control and HD cortex tissue, which is in accordance with previous studies [18, 20]. However compared with these studies, we detected more larger N-terminal htt fragments. Possibly, this is due to the use of different subject-cohorts or western blotting protocols. In our striatal samples, the molecular weight of the most prominent N-terminal htt fragment differed between HD and controls. This suggests a difference in htt proteolytic cleavage in striatum between controls and HD. This is in agreement with [18] although Mende-Mueller et al reported N-terminal htt fragments of smaller sizes. We were probably not able to show these smaller N-terminal htt fragments due to the weak htt western blot signal obtained for HD striatal tissue. El-Daher et al, who utilized the 4C8 antibody also observed weaker htt signals for HD striatum compared with controls [33]. On the other hand, studies using an anti N17 antibody did not observe a difference in htt signal between control and HD striatum. [18, 20]. Hence, the observed difference in htt western blot signal between control and HD and cortex versus striatum could be due to variability between different human post mortem brain tissue specimens.

## Conclusions

By mimicking PMD and a freeze-thaw cycle separately, we were able to provide a preliminary overview of expected effects on N-terminal htt fragments in post mortem human brain tissue. According to our results, PMD has a mild qualitative influence on N-terminal htt fragments because it introduces new fragments. A freeze-thaw cycle has a quantitative effect as it affects huntingtin signal strength. Although some reservations have to be taken with respect to extrapolating our results as we were only able to perform our PMD experiment on one control and one HD subject, our study contributes to the correct interpretation of data on N-terminal htt fragments obtained from human post mortem brain tissue. In addition, we have demonstrated a simple PMD mimicking protocol to be used on individual brain specimens. Furthermore, our results show that subject to subject variation has a larger, both qualitative and quantitative effect on N-terminal htt fragments. Hence, our study underscores the need for larger subject cohorts in studies involving post mortem human brain tissue.

## Supporting information

S1 Fig Levels of N-terminal huntingtin fragments in cortex and striatal tissue. All western blots shown at the same sensitivity. **(a)** Control subjects, Cortex region. **(b)** HD subjects, Cortex region. **(c)** Control subjects, Striatal region. **(d)** HD subjects, Striatal region. Control and HD subjects are age, sex and PMD matched. Upper blot: full length htt (fl htt). Middle blot: N-terminal htt fragments. Lower blot: β-actin. Post mortem delay in hours (hr).
(TIF)

S2 Fig N-terminal huntingtin fragments in control temporal cortex tissue from an individual subject including time points 24hr and 28hr. Western blot of Fig 1b with the additional timepoints T = 24hr and T = 28hr (underscored). The overall western blot signal for N-terminal fragments is slightly less.
(TIF)
