# Supplementary figures and images for: Effect of post-mortem delay on N-terminal huntingtin protein fragments in human control and Huntington disease brain lysates
Source: PLoS One. 2017 Jun 1;12(6):e0178556. doi: 10.1371/journal.pone.0178556 (PMC5453542; doi:10.1371/journal.pone.0178556)

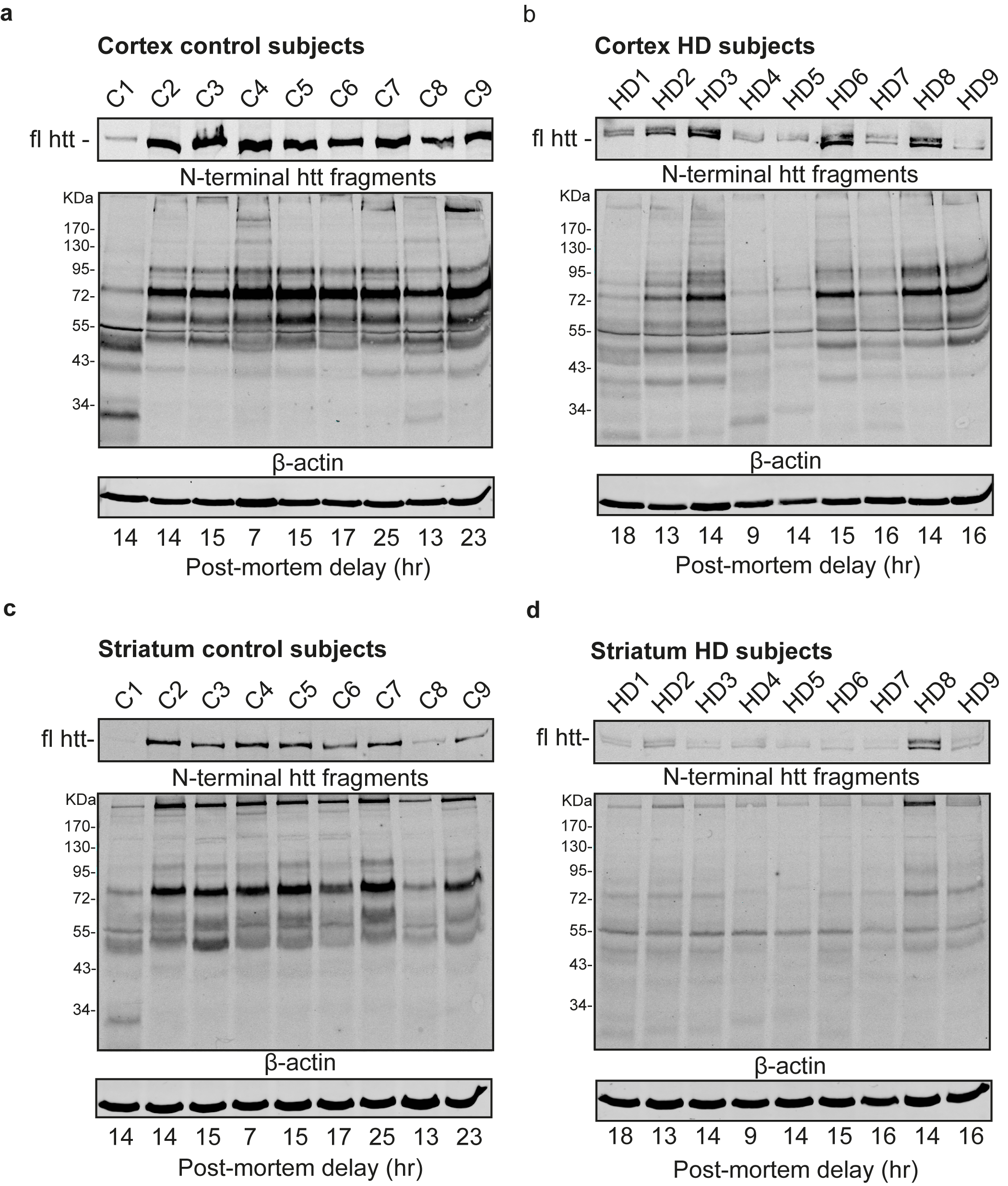

Supplement: S1 Fig — All western blots shown at the same sensitivity. (a) Control subjects, Cortex region. (b) HD subjects, Cortex region. (c) Control subjects, Striatal region. (d) HD subjects, Striatal region. Control and HD subjects are age, sex and PMD matched. Upper blot: full length htt (fl htt). Middle blot: N-terminal htt fragments. Lower blot: β-actin. Post mortem delay in hours (hr). (TIF) [file pone.0178556.s001.tif]

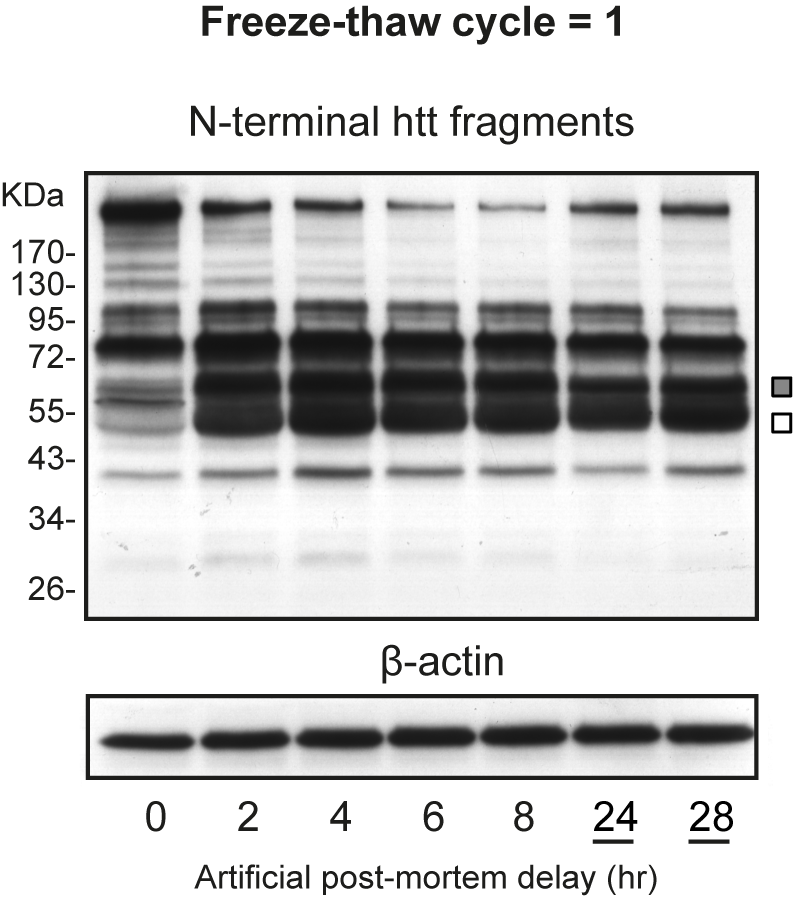

Supplement: S2 Fig — Western blot of Fig 1b with the additional timepoints T = 24hr and T = 28hr (underscored). The overall western blot signal for N-terminal fragments is slightly less. (TIF) [file pone.0178556.s002.tif]
